# Health professionals’ perceptions of palliative care for end-stage cardiac and respiratory conditions: a qualitative interview study

**DOI:** 10.1186/s12904-021-00805-3

**Published:** 2021-07-07

**Authors:** Hannah J. Fairlamb, Fliss E. M. Murtagh

**Affiliations:** 1 Hull York Medical School, University of Hull, Hull, UK; 2 Wolfson Palliative Care Research Centre, Hull York Medical School, University of Hull, Hull, UK

**Keywords:** Palliative Care, Hospice and Palliative Care Nursing, Advance Care Planning, Cardiology, Respiratory Tract Diseases

## Abstract

**Background:**

End-stage cardiac and respiratory diseases are common in the UK. People with these end-stage conditions experience similar, or even worse, symptomatic suffering to cancer patients but are less likely to receive specialist palliative care services. The objective of this study is to explore health professional perceptions and current practices in relation to specialist palliative care for patients with end-stage cardiac and respiratory disease.

**Methods:**

Qualitative study using in-depth interviews with health professionals, audio recorded and transcribed verbatim for thematic analysis. The study was conducted with doctors and nurses from cardiology, respiratory, and palliative care specialities in the UK. The participants had to be involved clinically in providing care to people with end-stage cardiac or respiratory diseases.

**Results:**

A total of 16 health professionals participated (5 cardiology, 5 respiratory, and 6 palliative care). Participants reported variable disease trajectories in these diseases making deciding on timing of palliative care involvement difficult. This was complicated by lack of advance care planning discussions, attributed to poor communication, and lack of health professional time and confidence. Participants reported poor interdepartmental education and limited specialist palliative care involvement in multidisciplinary teams.

**Conclusions:**

Palliative care for end-stage cardiac and respiratory diseases needs more attention in research and practice. Better integration of advance care planning discussions and early patient education/professional awareness are needed to enable timely referral to palliative care. Moreover, increased interdepartmental working for health professionals via joint educational and clinical meetings is perceived as likely to support earlier and increased referral to specialist palliative care services.

## Background

Chronic heart failure (CHF) and end-stage respiratory diseases are common chronic conditions in the UK and globally, with increasing prevalence [1, 2]. UK prevalence rate in 2018–19 for chronic heart failure was 920,000 [2]. The prevalence of CHF worldwide is currently estimated at 26 million, with millions of undiagnosed cases [3]. UK prevalence rates, in 2017–18, for chronic obstructive pulmonary disease (COPD) have been reported as affecting around 3 million people, with 2 million of these being undiagnosed [4]. It is estimated that the number of COPD cases globally in 2010 was 384 million, with a prevalence of 11.7%. These estimates are expected to rise significantly over the next 30 years [5]. Comparing mortality, cancer accounted for 28.1% of deaths in the UK in 2017, circulatory diseases account for 25%, and respiratory diseases for 13.8% [6]. Cardiovascular diseases are the number one cause of death worldwide, with over 17 million annual deaths [7]. This represents 31% of all global deaths [7]. With improving medications and interventions for management of cardiovascular diseases, CHF is a major and growing public health problem [4]. Worldwide in 2015, approximately 3.2 million deaths were due to COPD, making COPD the fourth leading cause of death [5]. It is estimated that globally by 2030 there may be over 4.5 million deaths annually from COPD and related conditions [5]. Patients with CHF and end-stage respiratory diseases experience significant symptomatic suffering and psychosocial burden, comparable to or worse than patients with advanced cancer [8–10]. Specialist palliative care may be as beneficial in these diseases as it is for cancer patients, with strong evidence supporting the benefits [8, 9, 11]. However, these non-cancer diseases are often referred to specialist palliative care later in the illness trajectory, or not at all [12, 13]. Suggested reasons include difficulty prognosticating, as well as limited knowledge and inaccurate perceptions of palliative care by health care professionals (HCPs) and patients [14, 15].

In recent years, the importance of palliative care in these diseases has been recognised and incorporated into UK guidelines. Current National Institute for Health and Care Excellence (NICE) UK guidance states that patients with CHF, COPD and other advanced lung diseases should have access to the full range of services offered by multidisciplinary palliative care teams. This includes admission to hospices, offered in a timely way, and appropriate to the patient’s needs and preferences [16–18]. Moreover, palliation for breathlessness and other symptoms, as well as advance care planning (ACP). Finally, addressing psychological, social, spiritual and family and carer needs are all recommended [16]. The NICE guidance also states that care should be consistent and effectively coordinated across all relevant settings and services [16]. This is intended to improve quality of life, reduce hospital admissions, and ensure a well-supported dying process.

This study therefore aims to explore health professional perceptions and current practices in relation to specialist palliative care for patients with end-stage cardiac and respiratory disease.

## Methods

### Study design

Qualitative study using in-depth interviews with health professionals to explore their perceptions of and current practices in referral to specialist palliative care for patients with end-stage cardiac and respiratory diseases.

### Sampling

Participants were purposively sampled by speciality (respiratory medicine, cardiology, and palliative medicine) and discipline (doctors and nurses). We use the term purposive sampling to mean selecting participants based on their knowledge and experience relevant to our study [19]. These were participants we felt could give us an in-depth understanding and insight into our research topics [19]. Purposive sampling of 2–4 doctors and 2–4 nurses from each of the three specialist areas was sought, with (overall) 6 to 8 from each discipline (doctors or nurses). A total sample of 12–16 participants was sought aiming for data saturation (no new themes), based on prior experience of similar work.

### Inclusion criteria

The inclusion criteria were doctors or nurses who work clinically in providing care to people with end-stage cardiac or respiratory diseases. Those with no direct involvement in clinical care of those with end-stage cardiac or respiratory diseases were excluded.

### Recruitment

Participants were accessed and invited via email through the existing networks of the Wolfson Palliative Care Research Centre at Hull York Medical School. Health professionals were given information about the study at time of invitation, and gave informed written consent before interview.

### Study development

An interview topic guide (Table 1) was developed based on the study objectives developed from a previously conducted literature review. This unpublished literature review was conducted by HF to gain greater insight into emerging themes and areas for research within the existing evidence. The topic guide was developed iteratively by the co-authors, and refined after comments from cardiology, respiratory medicine and palliative care colleagues. This set out to explore current practices and also perspectives on facilitators and barriers of referral to specialist palliative care.

**Table 1:** Interview topic guide

Interview topic	Areas covered
Participant experiences of referral of patients with advanced non cancer disease to palliative care	Deciding who to refer Judging time of referral Raising the topic of referral Approaching the topic of palliative care Discussing prognosis with patient Patient awareness of prognosis and palliative care
Participant experiences of barriers to palliative care referral	Difficulties in deciding when to refer to palliative care Other factors negatively influencing this Reasons for later referral for non cancer diseases
Participant experiences of facilitators to palliative care referral/involvement	Benefits from palliative care referrals Opinions on referring earlier Current timing of referrals Improving timing of referral to palliative care

### Data collection

All interviews were conducted between September and December 2019 by HF in person at the participant’s referred location (all in their place of work). The interviews were audio recorded and transcribed verbatim for analysis. The interviews were anonymised at the time of transcribing.

### Analysis

Interviews were thematically analysed [20, 21] by using the qualitative data analysis software NVivo 12 (B. Edhlund and A. McDougall). The process of thematic analysis started with HF transcribing the interviews and thoroughly reading through the data. Following this HF generated initial codes from the transcripts, identifying the themes from each interview. A coding tree was developed, iterated and refined from the data, and then applied, and further refined with the subsequent interviews [20, 21]. This was supported and reviewed with FM, to ensure codes were applied appropriately and consistently. The storage of the interview recordings and transcripts followed the data policy and guidance from the University of Hull.

### Ethics

Ethics approval was obtained from Hull York Medical School Research Ethics committee, Reference number: 19 25, Date of approval: 9 August 2019.

## Results

A total of 16 participants were interviewed; 10 female, 6 male. Their experience as health professionals ranging from one year to over 30 years. There were 5 cardiology, 5 respiratory, and 6 palliative care professionals (6 nurses and 8 doctors). Participants included 3 cardiology consultants, 1 cardiology speciality trainee, 1 specialist heart failure nurse, 3 respiratory consultants, 2 specialist respiratory nurses, 4 specialist palliative care nurses, and 2 palliative care consultants.

### Specialist palliative care involvement

Participants reported it was often difficult to decide when to involve specialist palliative care in end-stage cardiac and respiratory disease. Suggested reasons for this included the variable disease progression making prognostication very difficult, particularly in COPD and CHF, as discussed below.

*“The non-cancer trajectory is far less predictable. There are dips along the way and you do not know when the last one is going to be.” Participant 2, Palliative care nurse*

*“The variability between [heart failure] patients means you are often not sure what the prognosis is… it’s a bit meaningless to say this is your prognosis because the range is so great, between 3 and 16 years say.” Participant 7, Cardiology doctor*

*“Respiratory disease in general prognosticating is very difficult, in COPD we are terrible at it.” Participant 9, Respiratory doctor*

Due to the variable prognosis, there were differing views on when palliative care should be discussed with a patient. Some participants thought discussions about palliative care should be initiated at diagnosis, whereas some participants reported that they do not start to think about palliative care involvement until the patient has started to deteriorate quickly or there is no further medical management available.

*“I don’t particularly discuss [the involvement of palliative care] with patients you know because I could see these patients for 10 or 15 years with stable heart failure left ventricular (LV) dysfunction so I don’t go into that with every patient because if there’s 15 years of potential good life you’ve put a down in their mind without much benefit so I only discuss it when they are deteriorating and I recognise I’m running out of drug options for them. A lot of the time patients deteriorate and we get them better so I’m thinking ... well … palliative care probably don’t have much to offer at this point but the trouble is that some people don’t respond and quite suddenly you realise my plan hasn’t worked and this patient actually is palliative now, it’s almost that running out of options that drives a decision sometimes.” Participant 5, Cardiology doctor*

Attitudes were expressed from a cardiology perspective that once a patient is perceived to be end of life, they become the least important. The focus on evolving treatment options and interventions has become even more pronounced. This has led to poor management of the dying process.

*“Our mentality [within cardiology is that] those patients that are dying become somewhat less important, because everyone now concentrates on the people that aren’t going to die and can get better” Participant 8, Cardiology doctor*

### Advance care planning

Participants almost all identified advance care planning (ACP) as vital for early or appropriate timing of specialist palliative care involvement. However, there were discrepancies in participants' opinions of whose role this was and at what point it should happen, so that participants felt ACP did not happen effectively for many patients. Conflicting views on whose role it is to provide ACP, alongside the presumption these discussions were already being had by someone else, were highlighted as barriers to earlier ACP discussions).

*“I kind of presume that [advance care planning] is already happening but as I say for a lot of patients I have seen that makes me suspect that we aren’t having those conversations early enough.” Participant 5, Cardiology doctor*

*“I have felt for some people it has been lacking on the [advance care planning] side of it, I think that should be addressed earlier. I’m not quite sure whether it is being done, or how frequently and if those issues are being addressed.” Participant 6, Cardiology doctor*

Participant 15, gave the view that it is anyone’s role who is in an appropriate position. It is also important to do it at a time right for the individual patient. Some participants also expressed that ACP discussions are currently happening too late for patients and that they should be happening earlier to better educate the patient and enable delivery of more holistic care*.*

*“Anybody’s [role to raise advanced care planning] who is in the appropriate position. People often think it’s the doctors job but I think it’s best done by the person that knows the patient best, and then if a patient raises it, it’s being responsive at the moment instead of waiting for a set time.” Participant 15, Palliative care doctor*

*“I have felt like on a number of occasions that actually earlier on in the process of somebody being diagnosed with severe LV dysfunction we should probably be having better input in terms of making an advance care plan, because we know these people are likely going to die of their heart failure and we know what symptoms they are going to get, and in my experience sometimes they still don’t seem to have had those conversations when they’ve got to that stage.” Participant 6, Cardiology doctor*

Other difficulties identified for enabling earlier ACP and palliative care discussions included time pressures and lack of resources, demonstrated by quotations below.

*“There’s no question that when you are busy, you’re in a clinic, you are rushing, you’ve got 12 patients to see and there’s only 10 or 15 minutes for each patient, it doesn’t give you the space to have those more holistic discussions.” Participant 5, Cardiology doctor*

*“I think some of us [health professionals] are probably better than others at advance care planning. I think it’s become more difficult because of the pressures on the service, so we have our waiting list times are significantly longer than they should be for new patient appointments and follow up appointments…” Participant 9, Respiratory doctor*

*“[About advance care planning conversations] Nobody has the time to spend with [patients], nobody has the time to communicate with them.” Participant 8, Cardiology doctor*

Further barriers identified for ACP included loss of continuity with familiar HCPs. Participants 8 and 9 demonstrate how not knowing or loss of continuity with HCPs affects building relationships with patients, making sensitive and complex discussions more challenging. This was thought to be further influenced by the patient’s willingness to have those conversations, as well as their own beliefs around their disease.

*“You can have a condition and come and see a doctor, you can wait 6 months to see a cardiologist and get 5 minutes with them… it’s completely soul destroying… you go and see someone and you don’t even know who they are.” Participant 8, Cardiology doctor*

*“[Due to pressures on the health service and loss of follow up appointments] you then lose that continuity with patients so then it’s much harder to develop these [advance care] plans over time. Some patients are very pragmatic, so will meet you for the first time and be happy to discuss these things and put a plan in place and will document it. Others really aren’t and actually it requires numerous appointments for them to get to the point where they understand you and really trust you and you have a good feel for where they are at and building relationships is much much harder, so I think that’s making things more difficult.” Participant 9, Respiratory doctor*

### Patient and public’s perceptions of palliative care

Some participants believe their patients do not perceive their illness as a terminal diagnosis and have the opinion they can be medically treated or cured, leading to them not asking about ACP or palliative care or not being willing to discuss these topics.

*“Patients with chronic conditions, especially COPD and heart failure do not recognise it as a life limiting illness, whereas if you told a patient they had cancer their view would be very different to if you told them they had COPD.” Participant 4, Specialist palliative care nurse*

*“I think with COPD and heart failure there’s always people that get better when you take them in, they don’t get back to baseline but they get out of hospital and people think they’ve done a good job...patients and families still want to have treatment, they don’t want to think about giving up because they got better last time.” Participant 14, Palliative care doctor*

Moreover, misconceptions about what palliative care is may deter a patient from being willing to discuss the topic. Participant 14 describes how many patients view the hospice as a place to die, contrary to the wide variety of services they offer.

“I think a lot of people aren’t aware [of what palliative and hospice care is] or only think you can come [to the Hospice] when you are dying… no matter how much we try and dispel that myth, it takes a patient to come and see it for themselves.” Participant 14, Palliative care doctor

Participant 13 discusses the influence of cultural beliefs on public perception of diseases like CHF and COPD. Some participants compared the public perception of cancer to COPD and how the public perceives COPD as self-inflicted, adversely affects people’s view of the disease.

*“I think cancer attracts a great deal of emotion and empathy, I mean with things like COPD people often feel like it is the patient’s fault, perhaps you know they smoked, so people perhaps don't have as much sympathy for them.” Participant 13, Respiratory doctor*

Participants reported on the impact of socioeconomic status on public perception of the disease. COPD being more prevalent in lower socioeconomic classes was thought to lead to people not appreciating the extent of the disease burden. This was also thought to affect charitable and government funding for these diseases. This participant goes on to compare public perceptions of cancer and COPD influenced by media and charitable support.

*“Cancer affects anyone and everyone whereas COPD does predominantly affect people in lower socioeconomic classes and I actually think that does have an influence over financial support of these services [and] charitable services.” Participant 13, Respiratory doctor*

*“A completely different media image is portrayed of these things as well. Any cancer charity gets a lot of celebrity support but you don't really see that so things like COPD. I don't think people really recognise the symptomatic suffering and psychological suffering that a lot of these diseases go through and I think it's driven by the government as well an awful lot of money and resources has been put into cancer care and I think that's again responding to cultural beliefs about supporting people with illnesses.” Participant 13, Respiratory doctor*

### Communication and education surrounding palliative care

Some participants expressed they were not aware of what is to offer from specialist palliative care and other specialist cardiac or respiratory services in their local area, hence they do not refer or make late referrals.

*“I don’t think I’m aware of all the different things than can be offered at the hospice… and I think it’s important we work more closely because I think a lot of patients are going to need that dual input, especially at the end of life.” Participant 13, Respiratory doctor*

*“I think people aren’t always aware of our service [as a specialist heart failure team], I think a lot of junior staff aren’t aware of what we do. Personally I’d like to know what services specialist palliative do offer and how we can work alongside them as a heart failure team.” Participant 16, Specialist heart failure nurse*

*“I don’t really know at what point the [specialist] palliative care team would feel it is appropriate to get involved… maybe communicating that to us and training us.” Participant 6, Cardiology doctor*

Participant’s proposed solutions to this were improved communications between specialities and develop interdepartmental teaching. The importance of a multidisciplinary team meeting (MDT) was widely recognised by participants, with some participants the lack of specialist palliative care input within these teams.

*“I think it would be useful to have [specialist] palliative care involved at [COPD MDTs] because all of our patients have palliative care needs. Not all of the patient’s needs need to be addressed by specialist palliative care services but by having a presence at those meetings, one everyone else starts learning about what palliative care has to offer over and above and people will also start to get a feel that they might have something additional to offer over what can be done by the other teams.” Participant 9, Respiratory doctor*

*“I think having some, even if it's only one, that we actually meet or have a joint educational event [specialist palliative care and respiratory] I think those things are really useful in terms of improving patient care by bettering networks between ourselves, and having better understanding about what each of us do so we can offer it all patients. I think it would enable healthcare professionals to get to know each other better which would have a knock-on effect in improving patient care.” Participant 13, Respiratory doctor*

Participant 3 identifies the blurred line between what generalist palliative care a specialty can offer a patient and when specialist palliative care involvement would be beneficial.

*“I think what would make it easier is a clearer definition of what we offer as a [specialist palliative care] service and greater awareness amongst the teams because you’ve got generalist palliative care which in theory all staff, all nurses, all grade of doctors should have a good knowledge base of general palliative care and specialist palliative care which is very sort of the thin end of the wedge, the people who are very symptomatic or in emotional distress or they’ve got uncontrolled symptoms, they should be referred there and I think that is where the lines are blurred.” Participant 3, Palliative care nurse*

This line was thought to be further blurred by the role of specialist cardiology and respiratory nurses. Participants stated that specialist nurses are hugely important in the communication between a specialism and specialist palliative care and are often the ones to refer to palliative care. Some participants believed since the specialist nurses’ job was palliative in nature, they were taking care of the patients’ palliative care needs, making the referrals and were well integrated with the specialist palliative care team.

*“So I don’t recall ever having referred to palliative care over a heart failure patient but perhaps we should be more often and I think because we have the heart failure specialist nurses and specialist heart failure team I sort of feel like they are already getting that specialist input in terms of the medical treatment and that specialist nurse input for a bit more support so I use it less in that sense, but I’m not sure that we always recognise early enough or I don’t know, I don’t know how good the heart failure nurses are at providing that information of look you are coming to the end stage of our life and exploring their emotional needs and wishes and stuff.” Participant 5, Cardiology doctor*

*“I say I’ve kind of made the assumption, maybe I should have checked myself, that all of the issues surrounding heart failure and the palliative nature of it will be addressed by those specialist nurses.” Participant 6, Cardiology doctor*

However, specialist nurses themselves when questioned in this topic talked about the huge strain on their resources due to the large number of patients in their care. They stated they do not have the time and resources to take on the role of ACP, psychological support or specialist palliative care. Moreover, some participants reported that specialist nurses are not integrated well with the specialist palliative team.

*“Well it’s time and resources, like everything really. If we had more of us [speciality respiratory nurses] then we could take on that role [of advance care planning] ourselves quite well, we know the disease and the condition and it’s something we would do ourselves if we could but given the time and resources sadly we don’t have it.” Participant 11, Specialist respiratory nurse*

*“[Specialist palliative care nurses] are not very involved with [specialist heart failure nurses]. They tend to respond to a specific request and see somebody but they aren’t integrated with the heart failure service.” Participant 7, Cardiology doctor*

Participant 3 expanded on the constraints on time and resources specialist nurses are under. This makes it difficult to educate and train other HCPs on what generalist palliative care they can provide themselves and when specialist palliative care would be beneficial for a patient.

*“We struggle to have the time to see people, and our training courses are always really well received and it’s just because they don’t have enough time, so financial constraints and time and resources, we can’t give people the good palliative care knowledge base to start off with.” Participant 3, Specialist palliative care nurse*

### How to implement change

Participants suggested a range of solutions to promote ACP and earlier palliative care involvement. Firstly through better interdepartmental communication through teaching and MDTs.

*“If cardiology had more formal education of those that interfaced with [specialist palliative care] that might improve their input.” Participant 5, Cardiology doctor*

*“Some education would be beneficial, maybe some training days about what specialist services are on offer within the trust or the community just so they’ve got the awareness of what is out there.” Participant 12, Specialist respiratory nurse*

*“I think it’s useful if [palliative care and respiratory] actually had joint educational meetings or clinical meetings because that helps to improve both the referral from our side and making sure that patients are getting the best care.” Participant 13, Respiratory doctor*

Secondly, that there should be earlier integration of ACP with a way to ensure it is happening effectively for all patients. Some participants suggested introducing a set criteria or indicator tool to encourage advance care planning, and some participants said this was already starting to happen in their department. Finally, there should be better education for HCP’s to recognise the signs as to when ACP should be addressed, described by participant 2.

*“Having a do not resuscitate (DNR) discussion is not the same as having an advance care plan and I think if you are having those DNR conversations, I think we should also at that time be having conversations that are more detailed care plan of what would you want to happen if instance, and have it all clearly documented out… [so] you already have a document that goes with their [DNR], which is like an advance care plan.” Participant 6, Cardiology doctor*

*“So it’s educating the clinicians on the ward to recognise the patient is probably in the last year of life and then looking at things like, advance care planning, [DNR discussions], where the patient wants to be, what the family dynamics are, what support can the family give, working to try and do all the right things around end of life care.” Participant 2, Specialist palliative care nurse*

Another identified area for improvement was psychological support. Psychological support for patients was consistently believed to be very poor or inexistent. Many attributed this to a lack of funding and awareness, and some directly compared this to palliative cancer services which often have a health professional dedicated to psychological support.

*“I would say non cancer patients do not have access as readily to psychological support, that the cancer patients do. So cancer patients have psycho oncology, but there is no respiratory psychiatrist and I think that is a huge gap which could manage anxiety, breathlessness management and that is a huge area they could really improve and actually maybe reduce acute admissions in my mind. And the same with heart failure patients, I think if they had psychological support it would massively improve things.” Participant 4, Specialist palliative care nurse*

Finally, participants recognised that over recent years there has been a cultural shift away from palliative care just being associated with cancer patients. Through the continuation of this shift and education of younger generations, this was felt to likely facilitate better initiation of advance care planning and involvement of palliative care.

*“I think it’s culture, I think it’s really culture. So, I think seeing lots of the new younger doctors coming through really understanding the wider process and really understanding having more complex conversations. I think the other thing for me around culture is also to do with the whole ethos, not just the medical and nursing culture, but the ethos of the whole hospital, so organisational as well. If end of life care and palliative care and good engagement around those things is encouraged earlier on, through education, through a good supportive system… it becomes less fearful thing and more integrated.” Participant 1, Specialist palliative care nurse*

## Discussion

This study documents professionals' perceptions of specialist palliative care for patients with end-stage cardiac and respiratory diseases. Participants wanted palliative care involvement and described many of its benefits, but recognised it was often left too late in the disease trajectory for patients to fully benefit.

Participants reported cultures and attitudes which hinder specialist palliative care referrals. One aspect discussed by participants was the increasing focus, especially within cardiology, on prolonging life, with a wide range of complex treatment options. This made a palliative approach more difficult, and often delayed a timely transition to palliative care [22]. Other evidence supports this finding, with palliative care being seen as a defeat or failure for some cardiology professionals [22, 23]. Some professionals in our study thought there was no need for palliative care for CHF patients as too few patients would benefit; which is supported by the work of Ziehm et al. [23]. There is also the belief amongst professionals, both in our study and other research, that palliative care is synonymous with last days of life, contradictory to current guidance that palliative care should be utilised based on patient’s needs over the last year of life (or longer) [16, 17]. Other recent evidence shows that lack of awareness of palliative care services prevent discussions about involvement of palliative care and referrals [23, 24]. This is made more challenging due to the difficulty prognosticating about patients with CHF and COPD, and that timing of ACP and palliative care discussions can be difficult if patients might live with that condition for many years [23, 24]. An important consideration when investigating ACP and palliative care discussions is the patient’s perspective of their own disease. Lack of knowledge and poor patient education of both the disease process and role of palliative care can influence how patients respond to such discussions. The participants, supported by other evidence, report that this makes patients less likely to initiate or engage in discussions about prognosis and palliative care and are less willing to accept their mortality [15]. Another consideration is a patient’s acceptance (or denial) of having a chronic illness as a barrier to having these conversations.

Lack of clarity as to whose role it is to refer to palliative care emerged as a major issue. There was sometimes an assumption that specialist nurses would undertake ACP. However, our findings demonstrate this may not always be feasible within their busy roles. There also needs to be explicit discussion between teams about what is undertaken by specialist nurses within cardiology and respiratory services, what is undertaken by doctors within those teams, and what falls to primary care, and to specialist palliative care. Previous evidence shows that good interdepartmental communications are needed to ensure patients are reviewed regularly and these discussions are happening [25]. A number of authors have reported the significant need for ACP and good communication in people with CHF and COPD, but reports show unmet palliative care needs and infrequent ACP discussions [26, 27]. Another explanation identified for this, both by our study and other evidence, is lack of professionals’ confidence and communication skills needed to initiate these discussions [25]. This is further complicated by public misconceptions about palliative care [15]. Moreover, patients are often unable to think about and express their preferences early enough [15].

Participants in this study also identified loss of continuity with a health professional, due to pressure on time and resources, as a barrier to ACP discussions. Patients are more likely to have difficult and challenging ACP discussions with a professional they know and trust [28]. Some participants believed specialist heart failure and respiratory nurses are best suited to this role. This concords with evidence that nursing staff tend to initiate palliative care earlier than the physicians [23]. The difficulties of advocating any one specific group of professionals to undertake these roles is recognised, and research suggests this needs to be well managed through effective communication and team working of the healthcare team alongside patient preferences [29].

The current NICE guidance advises timely involvement of palliative care coordinated effectively across all relevant settings and services [17, 18]. However, the participants reported this is not happening effectively and there is a need for better interdepartmental communications and MDTs to discuss needs of patients and ensure ACP is occurring. Interdepartmental education and training to inform professionals of what local services are available, what specialist palliative care can offer patients and when this is appropriate is needed [25].

Research recommends all professionals involved in the care of patients with CHF and COPD should have good generic palliative and communication skills [29]. Also, “complex or persistent problems (e.g. intractable pain, difficult breathlessness)” should trigger referral to specialist palliative care services and this is not only for the dying patient [29]. Some participants recognised that there has been a small cultural shift towards earlier involvement of ACP and palliative care in these conditions, but this change in culture needs to be continued [25].

## Limitations

This was a small sample of 16 HCPs, with only doctors and nurses being included, excluding other HCP roles. We acknowledge that social workers, allied and other health professionals will provide useful additional insights; their perspectives could be the focus of future research. However, our focus on the perspectives of doctors and nurses did allow for the consistency of specific themes to be explored, and no new themes emerged from the later interviews. We also acknowledge that there were few participants from community settings and no involvement of general practice; however, the focus here was specifically on specialist palliative care referral, rather than generalist end of life care (where primary care teams often play the major role). Participants were also recruited through the Research Centre networks, which may have led to a sample of those more engaged with research, and with implementation of research into practice. We consider however, that this does not undermine our findings, which emerged reasonably consistently across specialities and disciplines. The sample was however concentrated in just one region of the UK; practices may differ across other regions.

## Conclusion

Better integration of ACP discussions earlier in the disease trajectory is needed to enable timely referral to palliative care. Barriers to this included uncertainty of whose role ACP (and referral) was, when it is appropriate to discuss, and professionals’ confidence and communication skills. The need for better interdepartmental working relationships between cardiology/respiratory and palliative care via joint education and clinical meetings is highlighted. A cultural shift needs to be made regarding public awareness of end-stage cardiac and respiratory diseases and the value of palliative care.

## Data Availability

The datasets generated and/or analysed during the current study are not publicly available due to the data policy and guidance from the University of Hull but are available from the corresponding author on reasonable request.

## Abbreviations

COPD: Chronic obstructive pulmonary disease
CHF: Chronic heart failure
ACP: Advance care planning
HCP: Health care professional
LV dysfunction: Left ventricular dysfunction
